# Impact of coronary flow on the risk of microvascular obstruction in acute myocardial infarction

**DOI:** 10.1186/1532-429X-18-S1-P93

**Published:** 2016-01-27

**Authors:** Nadine Abanador-Kamper, Lars Kamper, Vasiliki Karamani, Patrick Haage, Melchior Seyfarth

**Affiliations:** 1 Department of Cardiology, Witten/Herdecke University, Helios Medical Centre, Wuppertal, Germany; 2 Department of Diagnostic and Interventional Radiology, Witten/Herdecke University, Helios Medical Centre Wuppertal, Wuppertal, Germany

## Background

Microvascular obstruction (MO) and coronary flow have been independently described to have a high prognostic impact in patients after acute myocardial infarction. The interdependence of these facts has not been elucidated, so far. Aim of this study was to investigate the impact of pre- and post-interventional coronary flow on the occurrence of MO in patients with acute myocardial infarction.

## Methods

336 patients with acute myocardial infarction were examined by cardiac magnetic resonance imaging after primary percutaneous coronary intervention. Patients were categorised into two groups based on the presence or absence of MO. Procedural characteristics and marker of infarct size were included in the analysis.

## Results

MO was present in 110 (33%) and absent in 226 (67%) patients. In the univariate comparison both groups differed significantly regarding age, sex, the qualifying event, pre- and post-interventional thrombolysis in myocardial infarction (TIMI) flow, as well as parameters of infarct size. However, after multivariable regression analysis pre-interventional TIMI-flow 0, proximal culprit lesion, post-interventional TIMI-flow <III and CK-MB remained strong independent predictors for MO. Odds ratios for these factors were 2.31 (95% CI 1.04-5.11, *P*=0.034) for pre-interventional TIMI-flow 0, for proximal culprit lesion 11.94 (95% CI 5.70-25.01, *P*<0.001), for post-interventional TIMI-flow III 0.28 (95% CI 0.10-0.74, *P*=0.010) and for CK-MB 1.50 (95% CI 1.24-1.82, *P*<0.001).

## Conclusions

Pre-interventional proximal coronary artery occlusion (TIMI 0) and insufficient post-interventional coronary reperfusion (TIMI-flow <III) have a high impact on the occurrence of MO in acute myocardial infarction. Furthermore patients with MO show significant higher markers of infarct size.

